# PDC-Net: A Prefrontal Dual-Channel Network with Gated Mamba Interaction for Cross-Subject Visual Attentional State Decoding

**DOI:** 10.3390/brainsci16090915

**Published:** 2026-08-27

**Authors:** Tianyuan Niu, Ruoyan Li, Mengfan Li

**Affiliations:** 1 School of Hebei Sciences & Biomedical Engineering, Hebei University of Technology, Tianjin 300130, China; 235490@stu.hebut.edu.cn (T.N.); 245727@stu.hebut.edu.cn (R.L.); 2 State Key Laboratory of Reliability and Intelligence of Electrical Equipment, Hebei University of Technology, Tianjin 300130, China; 3 Tianjin Key Laboratory of Bioelectromagnetic Technology and Intelligent Health, Hebei University of Technology, Tianjin 300130, China; 4 Hebei Key Laboratory of Bioelectromagnetics and Neuroengineering, Hebei University of Technology, Tianjin 300130, China

**Keywords:** electroencephalography, visual attentional state decoding, prefrontal dual-channel EEG, mamba, brain–computer interface

## Abstract

**Highlights:**

**What are the main findings?**

- PDC-Net achieves 76.76% cross-subject accuracy in distinguishing Visual Sustained Attention (VSA) and Visual Internally Directed Cognition (VIDC) states using only two forehead EEG channels, Fp1 and Fp2.
- The TRAB and CGMI modules capture complementary temporal and cross-branch features and contribute to decoding performance under subject-independent evaluation.

**What are the implications of the main findings?**

- The dual-channel configuration reduces EEG acquisition requirements and provides a basis for future wearable-system evaluation.
- PDC-Net achieves a favorable accuracy–computational-cost trade-off under the evaluated offline GPU setting.

**Abstract:**

**Background/Objectives:** Electroencephalography (EEG), with its high temporal resolution, non-invasiveness, and cost-effectiveness, provides a suitable modality for investigating task-related signal patterns associated with Visual Sustained Attention (VSA) and Visual Internally Directed Cognition (VIDC), but cross-subject generalization remains challenging in reduced-channel settings. This study evaluated PDC-Net under an offline GPU setting. **Methods:** PDC-Net uses a Temporal Representation Adaptation Block (TRAB) for local temporal transformation and feature-channel recalibration and a Cross-Branch Gated Mamba Interaction (CGMI) module for input-dependent bilateral information exchange and long-range sequence modeling. **Results:** Under the strict LOSO cross-validation setting, PDC-Net achieved an average decoding accuracy of 76.76%, representing the highest mean accuracy among the 11 evaluated models. The model also maintained a favorable balance between cross-subject decoding performance and computational cost under the evaluated offline GPU setting. **Conclusions:** These findings support the feasibility of offline, subject-independent VSA/VIDC decoding from dual-channel Fp1/Fp2 EEG under the evaluated controlled conditions and provide a basis for subsequent external, cross-session, online, and hardware-specific evaluation.

## 1. Introduction

Attention is a fundamental component of higher-order cognition and is essential for information processing, decision-making, and behavioral regulation [1,2]. Accurate monitoring of attentional states is therefore important in educational assessment, human–computer interaction, and safety-critical environments [3]. Among available physiological signals, electroencephalography (EEG) is particularly suitable for continuous attention monitoring because of its millisecond-level temporal resolution and ability to capture rapid neural dynamics [4,5]. However, robust cross-subject decoding remains difficult, especially in wearable systems with only a few electrodes, where limited spatial information and pronounced inter-subject variability must be addressed simultaneously. In practical applications, the objective is often to monitor sustained changes in cognitive engagement under continuous and relatively naturalistic conditions rather than transient selective-attention responses elicited by paradigms such as Oddball or Posner cueing tasks. Continuous paradigms have consequently been adopted for cognitive-state monitoring [6,7], and recent studies have demonstrated the feasibility of distinguishing sustained externally and internally directed states from EEG dynamics during continuous visual stimulation [8].

To achieve EEG-based attention decoding, early studies primarily relied on traditional feature engineering methods, extracting time-domain statistical features, frequency-domain power spectral density, and time–frequency representations, followed by classifiers such as support vector machines (SVM). For instance, Klimesch systematically investigated the relationship between spectral power variations and cognitive processes, demonstrating the importance of alpha and theta rhythms in attentional modulation [9]. Huang et al. employed independent component analysis (ICA) to uncover latent neural sources associated with cognitive tasks, providing a foundation for feature extraction [10]. These studies demonstrated that EEG contains attention-related information, although the performance of conventional pipelines may depend on the selected handcrafted features. Deep-learning methods have therefore been increasingly investigated as data-driven alternatives for EEG decoding [11,12,13]. More recently, Transformer-based models have been introduced into EEG analysis to capture long-range dependencies through self-attention. Under reduced-channel conditions, convolutional models may be limited in representing global bilateral interactions, whereas Transformer-based approaches generally introduce greater computational and memory requirements. Consequently, existing methods struggle to balance representational power and computational efficiency under minimal-channel constraints, limiting their practical applicability [14].

A further challenge is the substantial inter-subject variability of EEG signals. Differences in neurophysiology, cognitive strategies, and recording conditions can produce distribution shifts across individuals [15], causing a model to learn subject-specific rather than generalizable representations. Domain adaptation and adversarial learning have been used to reduce cross-subject feature discrepancies [16,17], whereas contrastive and self-supervised learning aim to obtain more transferable EEG representations [18,19,20]. Although these approaches have improved cross-subject performance, they may introduce additional training or computational complexity. Learning discriminative and subject-robust representations within a compact architecture, therefore, remains an important challenge for wearable EEG decoding.

From a neurophysiological perspective, sustained external attention and internally directed cognition both involve top-down regulation supported by the prefrontal cortex (PFC) [1,2]. Under the international 10–20 system, Fp1 and Fp2 provide a bilaterally symmetric frontopolar recording configuration. Frontal EEG activity contains theta-, alpha-, and beta-band dynamics associated with attentional control, cognitive engagement, and alertness regulation [5,21,22]. In particular, the balance between slower theta activity and faster beta activity has been investigated as an electrophysiological correlate of attentional control [21,22]. Retaining Fp1 and Fp2 as separate branches preserves bilateral information while allowing coordinated temporal activity to be modeled without the acquisition burden of a high-density montage. Because Fp1 and Fp2 are scalp recording locations, they should not be interpreted as anatomically specific measurements of individual prefrontal sources. Instead, this configuration provides a practical compromise among bilateral information preservation, electrode-placement requirements, and compatibility with reduced-channel systems.

Based on the above considerations, we propose PDC-Net, a compact dual-branch network for cross-subject decoding of Visual Sustained Attention (VSA) and Visual Internally Directed Cognition (VIDC) from Fp1/Fp2 EEG. The two channels are initially processed in separate branches to preserve channel-specific information. A Temporal Representation Adaptation Block (TRAB) performs local temporal feature transformation and feature-channel recalibration, whereas a Cross-Branch Gated Mamba Interaction (CGMI) module models coordinated bilateral dynamics using linear-complexity state-space operations [23]. The main contributions are as follows:

(1) A compact hierarchical TRAB is developed to transform and adaptively recalibrate task-relevant temporal patterns from minimal-channel EEG;
(2) A gated Mamba-based CGMI module is introduced to model long-range coordination between the two frontopolar recordings while preserving branch-specific information;
(3) The resulting framework is evaluated under a strict Leave-One-Subject-Out protocol, demonstrating the feasibility of cross-subject VSA/VIDC decoding using only Fp1 and Fp2.

## 2. Methodology

**Figure 1:**
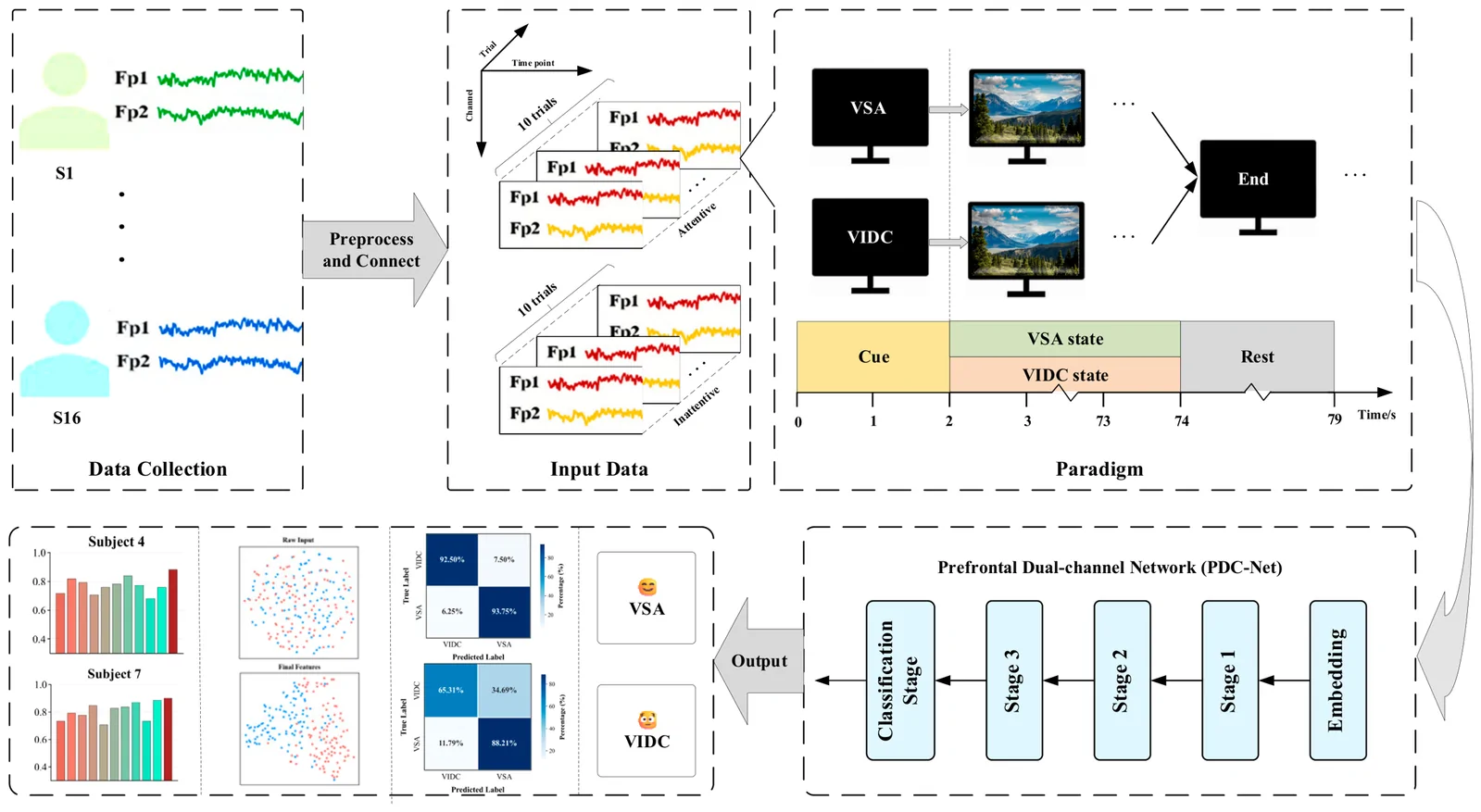
Overall end-to-end pipeline of this study.

Figure 1 illustrates the overall framework covering dual-channel EEG acquisition, preprocessing, and paradigm-driven VSA/VIDC state decoding. PDC-Net adopts a “divide-and-conquer then interact” strategy with three hierarchical stages. Separate Fp1 and Fp2 branches first encode channel-specific signals. Three hierarchical stages then apply TRAB-based temporal transformation and feature-channel recalibration, followed by CGMI-based cross-branch interaction. The resulting branch representations are aggregated and passed to the classification head.

### 2.1. Dual-Channel Independent Temporal Embedding

To preserve channel functional asymmetry for VSA/VIDC state decoding, PDC-Net employs a dual-branch temporal embedding module that independently processes left (Fp1) and right (Fp2) EEG signals. Instead of early fusion, two parallel streams $X_{L}$ and $X_{R}$ are projected through non-shared parameters $\theta_{L}$ and $\theta_{R}$:

(1) $$H_{L}=E_{stem}^{L}\left(X_{L},\;\theta_{L}\right)$$

(2) $$H_{R}=E_{stem}^{R}\left(X_{R},\;\theta_{R}\right)$$

This design preserves channel-specific temporal information before cross-branch interaction. Temporal feature extraction is initially performed using a one-dimensional convolution with a kernel size of 3, corresponding to a local three-sample receptive field at the 250 Hz sampling rate. This operation is interpreted as a local encoder of short-duration waveform patterns rather than as a frequency-band-selective filter. The subsequent hierarchical TRAB and CGMI stages progressively enlarge the effective receptive field and model broader temporal dependencies. Gaussian Error Linear Unit (GELU) activation [24] and Batch Normalization (BN) [25] are applied after convolution. The module maps the raw EEG signals to a 32-dimensional latent representation while preserving their temporal organization.

### 2.2. Temporal Representation Adaptation Block

To enhance feature quality under noisy EEG conditions, TRAB combines convolution-based temporal feature transformation with feature-channel recalibration. Specifically, the pointwise and depthwise convolutions transform local temporal patterns, whereas the squeeze-and-excitation operation [26] derives one recalibration weight for each expanded feature channel from globally pooled temporal statistics. Therefore, TRAB does not compute an attention distribution over individual temporal positions. The block adopts a residual bottleneck structure [27] to support hierarchical feature extraction and stable gradient propagation. The input is first expanded using a pointwise convolution:

(3) $$X_{\mathrm{expand}}\;=\;\mathrm{Dropout}(\mathrm{GELU}(\mathrm{BN}({Conv}_{1\times 1}(X_{in}))))$$

The expanded representation is subsequently transformed using a single depthwise convolution with a kernel size of 15, a stride of 1, a dilation rate of 1, and a padding size of 7:

(4) $$X_{dw}=\;GELU(BN(DW{Conv}_{k=15,s=1,d=1,p=7}(X_{expand})))$$

The pointwise convolution expands the intermediate feature dimension before depthwise transformation. Global temporal statistics $z_{c}$ are then computed for feature-channel recalibration:

(5) $$z_{c}\;=\;\frac{1}{T}\sum_{t=1}^{T}X_{\mathrm{dw}}(c,\;t),\;z\;=\;[z_{1},z_{2},\ldots ,\;{zC}_{mid}{]}^{T}$$

Channel-wise weights are generated via a bottleneck MLP:

(6) $$s\;=\;\sigma {(W}_{2}\cdot \mathrm{ReLU}{(W}_{1}\cdot z))$$

Each recalibration weight is shared across all temporal positions within its corresponding feature channel. The weighted features are projected to the original feature dimension and combined with the residual connection:

(7) $$X_{\mathrm{out}}\;=\;\mathrm{BN}\left({\mathrm{Conv}}_{1\times 1}\left(X_{\mathrm{dw}}\;⊗\;s\right)\right)+X_{in}$$

By stacking TRAB modules across multiple stages, the network progressively enlarges its effective temporal receptive field while recalibrating feature-channel responses. Accordingly, TRAB is interpreted as a temporal feature transformation and feature-channel recalibration module rather than an attention mechanism that assigns weights to individual time points.

### 2.3. Cross-Branch Gated Mamba Interaction

While TRAB focuses on intra-branch feature refinement, modeling inter-channel interaction is essential for capturing coordinated neural activity [17]. To this end, the CGMI module leverages a State Space Model (SSM) for efficient long-range dependency modeling. Following the standard Mamba formulation, the continuous-time SSM is discretized using a zero-order hold (ZOH) scheme before being applied to sampled EEG sequences. Specifically, given a continuous-time system parameter pair (*A*, *B*) and an input-dependent step size *Δ*, the discretized transition matrices $\bar{A}$ and $\bar{B}$ are formulated as:

(8) $$\bar{A}=\;e^{\Delta A}$$

(9) $$\bar{B}=(e^{\Delta A}-I)A^{-1}B$$

**Figure 2:**
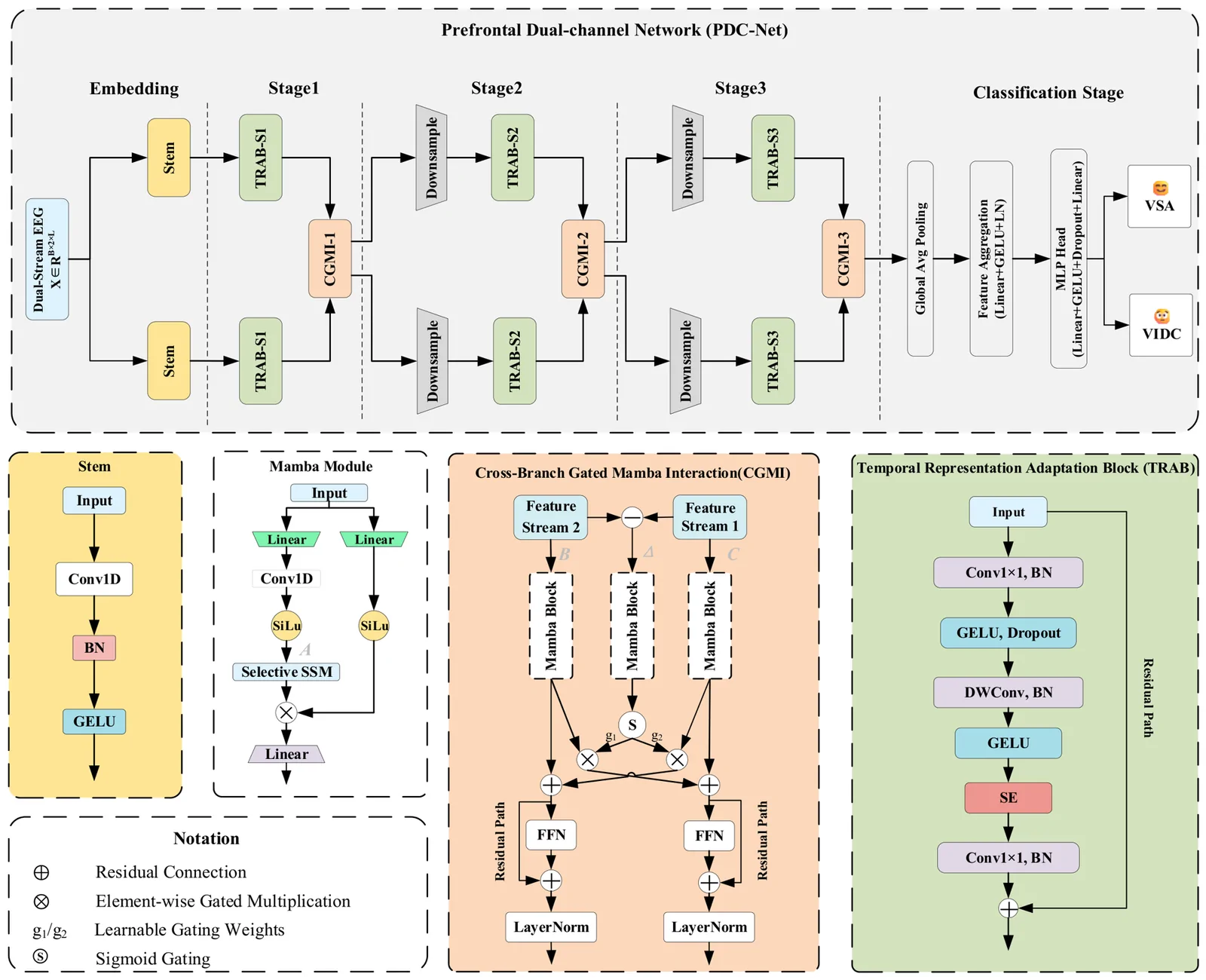
The flowchart of the proposed method.

As illustrated in Figure 2, the CGMI module is embedded within each hierarchical stage to enable progressive cross-branch information exchange. Each CGMI unit contains three independent Mamba blocks: two for processing single-branch features and one dedicated to generating cross-branch gating signals. By leveraging these discretized matrices, the Mamba block computes the discrete-time latent state transitions and system outputs at time step *t* as [28]:

(10) $$h(t)\;=\;\bar{A}h(t-1)+\bar{B}x(t)$$

(11) y(t)=Ch(t)

where input-dependent parameters enable adaptive temporal filtering based on the current input.

At Stage *i*, each CGMI unit contains three independent Mamba blocks with non-shared parameters: two blocks process the Fp1 and Fp2 branch representations separately, whereas the third block processes their feature difference to generate the cross-branch gates. Each Mamba block uses *d*_model_ = *D_i_*, *d*_state_ = 16, *d*_conv_ = 4, an expansion factor of 2, and an automatically determined *dt*_rank_ = [*D_i_*/16] [23]. A single selective scan is performed in chronological order without an additional reverse scan; thus, the Mamba component is unidirectional rather than bidirectional. Nevertheless, the complete PDC-Net is implemented as an offline, non-causal window classifier because the centered temporal convolutions and global pooling operation use information from the complete 3 s input segment.

To enable cross-branch interaction, the two Mamba-processed branch outputs are first compared through their feature difference. This difference is then processed by the third Mamba block and a linear-sigmoid projection to generate the adaptive gates $g_{1}$ and $g_{2}$, which dynamically regulate information exchange:

(12) $$g_{1},g_{2}\;=\;\sigma \left(\mathrm{Linear}\left({Mamba}_{gate}(H_{Mamba1}\;-\;H_{Mamba2})\right)\right)$$

These time-varying gates dynamically control the information flow between branches. The gated features from the opposing channel are injected into each stream to produce bilateral, context-aware representations:

(13) $${\hat{H}}_{1}=\left(g_{1}\;⊗\;H_{Mamba2}\right)+H_{Mamba1}$$

(14) $${\hat{H}}_{2}=\left(g_{2}\;⊗\;H_{Mamba1}\right)+H_{Mamba2}$$

where ⊗ denotes element-wise multiplication. This operation allows each branch to incorporate input-dependent information from the opposite branch. The resulting representations are then refined by an FFN:

(15) $$H_{out1}=LayerNorm({\;\hat{H}}_{1}+{FFN}_{1}({\hat{H}}_{1})\;)$$

(16) $$H_{out2}=LayerNorm({\;\hat{H}}_{2}+{FFN}_{2}({\hat{H}}_{2})\;)$$

Applying CGMI after each TRAB stage enables progressive cross-branch interaction throughout the network.

### 2.4. Feature Aggregation and Classification Head

The two Stage 3 branch representations have the same shape, (*B*, *L*_3_, *D*_3_), and are first combined through element-wise addition. In the implementation, Align denotes a parameter-free permutation from (*B*, *L*_3_, *D*_3_) to (*B*, *D*_3_, *L*_3_), which converts the sequence-first representation into the channel-first format required by AdaptiveAvgPool1D. Align does not perform temporal interpolation or learnable feature projection. The aggregation is formulated as:

(17) $$V_{\mathrm{global}}\;=\;\mathrm{GAP}\left(\mathrm{Permute}((B,L,D)\;\to \;(B,D,L))({\hat{H}}_{Stage3}^{1}\;+\;{\hat{H}}_{Stage3}^{2})\right)$$

Global Average Pooling (GAP) subsequently aggregates the 188 temporal positions into a 128-dimensional representation [29]. The aggregated feature is further refined:

(18) $$V_{\mathrm{fc}}\;=\;\mathrm{LayerNorm}\left(\mathrm{GELU}(\mathrm{Linear}(V_{global}))\right)$$

Final predictions are obtained via:

(19) $$\hat{y}\;=\;{Linear}_{2}({\mathrm{Dropout}}_{0.2}(GELU({Linear}_{1}(V_{fc}))))$$

Dropout (0.2) is applied between the two linear layers of the classification head for regularization [30]. This design aggregates information across the complete 3 s segment before classification.

### 2.5. Implementation and Training Objective

To optimize the cross-subject decoding network, all experiments were conducted on dual-channel prefrontal EEG signals with an input shape of (*B*, 2, *T*), where *B* denotes batch size, and *T* denotes the number of time steps. PDC-Net is trained end-to-end using the standard categorical cross-entropy loss function, which quantifies the discrepancy between the predicted attention state distributions and the true labels.

(20) $$L_{CE}=-\frac{1}{N}\sum_{i=1}^{N}\sum_{j=1}^{K}y_{i,j}\log({\hat{y}}_{i,j})$$

where *N* is the batch size, and *K* = 2 corresponds to the two visual attentional states.

Optimization was performed using Adam [31] with weight decay for regularization. Within each outer LOSO fold, the initial learning rate was selected from {1 × 10^−4^, 5 × 10^−4^, 1 × 10^−3^}, and weight decay was selected from {0, 1 × 10^−4^, 1 × 10^−3^}, exclusively according to validation accuracy. Gradient clipping with a maximum norm of 1.0 was applied to stabilize training. The model was trained for a maximum of 100 epochs with a batch size of 32, and the learning rate was reduced by a factor of 0.1 when validation loss did not improve for 10 consecutive epochs. The complete optimization search space is reported in Supplementary Table S5.

The detailed layer configuration of PDC-Net is provided in Table 1.

## 3. Experiment

### 3.1. Experimental Paradigm Design

**Figure 3:**
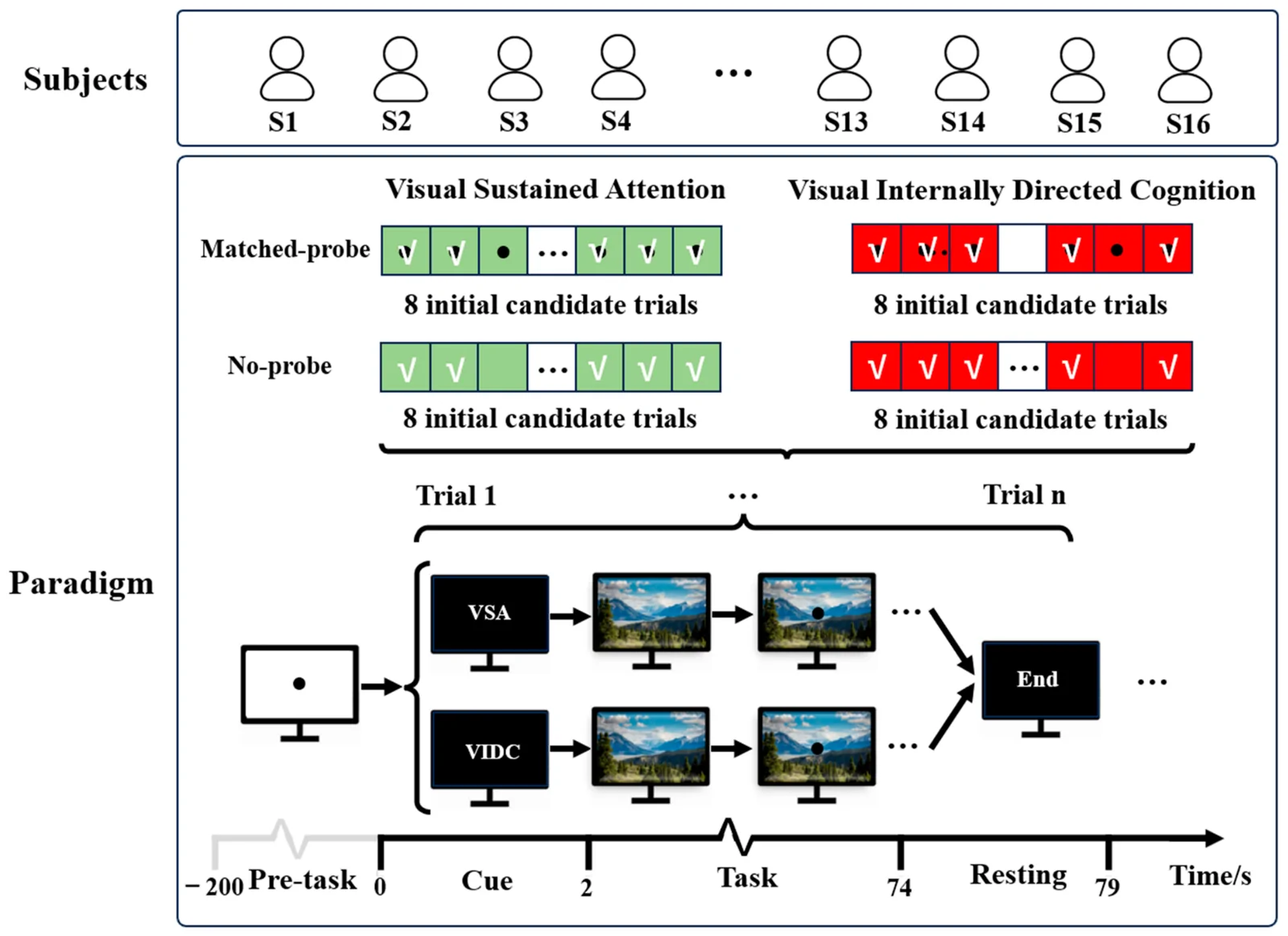
Timeline of the task paradigm. The diagram shows the initial candidate-trial block. Additional VSA–VIDC paired trials were collected only when fewer than five eligible pairs remained in a probe condition. Black dots denote visual probe events, white check marks identify trials retained for analysis, blank boxes indicate candidate trials that were not retained, and ellipses denote omitted repeated participants or trials.

To investigate the neural correlates of externally and internally directed attentional states while minimizing stimulus-related confounding factors, the present study employed a consistent-stimuli experimental paradigm in which identical visual stimuli were presented across conditions, with only task instructions differing. Specifically, participants completed two experimentally instructed attentional conditions: a VSA condition, in which they continuously observed the image on the screen, and a VIDC condition, in which they maintained the same visual environment while refraining from intentional monitoring and allowing thoughts to unfold naturally. It should be noted that the VIDC condition does not represent spontaneous mind wandering but rather an experimentally instructed internal cognitive state designed to facilitate internally directed cognition under controlled laboratory conditions [32,33]. To dissociate the attentional manipulation from probe-related effects, the experiment adopted a 2 × 2 within-subject factorial design comprising attentional state (VSA vs. VIDC) and probe condition (matched-probe vs. no-probe). This resulted in four trial types: VSA with matched probes, VIDC with matched probes, VSA without probes, and VIDC without probes. Within each probe condition, the visual input and trial structure were identical between VSA and VIDC, such that the attentional instruction constituted the only systematic difference between the two states. A detailed overview of the experimental procedure is illustrated in Figure 3.

Eight static natural-scene images were selected as visual stimuli, all depicting neutral natural landscapes without humans, animals, buildings, text, vivid colors, or salient motion-related elements, and standardized in resolution, luminance, and contrast to reduce low-level variability and minimize oculomotor confounds such as gaze shifts and exploratory scanning. The images were obtained from the Unsplash Dataset under the applicable access and licensing conditions. The use of neutral natural scenes was consistent with established natural-scene perception paradigms [34], and the images were obtained from the Unsplash Dataset [35]. Each participant initially completed 32 candidate trials, comprising eight trials for each of the four combinations of attentional state and probe condition. Trial retention was performed at the paired level within each probe condition: a VSA–VIDC pair was eligible only when both trials sharing the same Pair ID satisfied the predefined behavioral and signal-quality criteria. Five eligible pairs were retained in the matched-probe condition and five in the no-probe condition, resulting in 20 analyzed trials per participant. When fewer than five eligible pairs remained among the eight initial pairs, additional paired trials were collected after a rest period until five eligible pairs were obtained. Three participants required additional acquisition, comprising nine paired sets and 18 individual trials; consequently, 530 candidate trials were collected across the cohort. Each of the eight images was presented once in each experimental combination during the initial trial block, and replacement-trial image assignments were counterbalanced to minimize image- and sequence-related effects.

Each trial had a nominal duration of 72 s, from which the available EEG analysis intervals were determined after probe-related and artifact-related exclusions. In each matched-probe trial, four visual probes were presented as brief target events, each defined as a small black circular dot displayed at the center of the screen for 0.5 s. No visual probes were presented during the no-probe trials. Probe schedules were generated in advance from a larger pool of pseudo-random schedules at an integer-second temporal resolution. Each schedule contained four probe onsets restricted to 8–66 s after trial onset, and consecutive probe intervals were constrained to 10–16 s. For each participant, every VSA matched-probe trial was paired with a VIDC matched-probe trial using exactly the same probe-onset schedule. Different trial pairs used different schedules, and the two trials sharing a schedule were presented non-consecutively. Schedule assignments were varied across participants to avoid a fixed temporal pattern across trials or participants. The corresponding schedule was used as a virtual-probe schedule for the paired no-probe trials during offline data extraction.

Matched-probe and no-probe trials were intermixed in a randomized and counterbalanced order. Participants were informed of the required attentional state before each trial but were not informed whether probes would appear during that trial. The no-probe condition was included to evaluate whether VSA/VIDC decoding remained possible in the absence of probe presentation, probe-related motor responses, and repeated task interruption. This arrangement also reduced the certainty that a probe would occur during any given trial, although it was not assumed to eliminate general temporal expectation completely. In matched-probe trials, participants responded to each probe as quickly and accurately as possible by pressing the “Y” key. EEG data within 3 s following each probe onset were excluded to reduce contamination from probe-evoked perceptual processing, attentional reorientation, and motor responses [36,37].

To ensure the validity of instructed attentional states and provide an independent behavioral measure of engagement, a probe-based behavioral quality-control procedure was implemented [38,39]. Prior to the experiment, each participant completed a ~200 s calibration session in which only probes were presented without background images, with fully pseudo-random inter-probe intervals ranging from 10 to 16 s, enabling estimation of baseline perceptual–motor response characteristics independent of visual scene processing. Individual-specific baseline measures of reaction time (RT), reaction time variability (RTV), and omission responses (lapses) were derived and used to construct behavioral reference distributions [40,41,42]. A common post-trial assessment was administered after all matched-probe and no-probe trials to evaluate external visual attention, internally directed cognition, and adherence to the instructed state. These ratings were obtained only after the 72 s EEG recording period and therefore did not interrupt the analyzed interval. For matched-probe trials, reaction time, reaction-time variability, and omission responses were additionally recorded and, together with the common post-trial ratings and signal-quality criteria, were used to determine trial validity according to predefined thresholds [38,39,40,41,42]. For no-probe trials, for which probe-based behavioral measures were unavailable, trial validity was determined using the same post-trial ratings and signal-quality criteria. Behavioral trial screening was completed before EEG segmentation and without reference to subsequent classification results. A trial was considered signal-quality eligible only when at least 16 artifact-free, non-overlapping 3 s candidate windows remained after actual- or virtual-probe-related exclusion and artifact inspection. Individual-trial eligibility was determined first, after which trials sharing the same Pair ID were evaluated jointly. A pair was eligible only when both its VSA and VIDC trials satisfied the applicable behavioral and signal-quality criteria. Five eligible pairs were retained in each probe condition. When the eight initial pairs did not provide five eligible pairs, additional paired trials were collected. Individual trials not included in the final dataset were classified as behaviorally excluded, signal-quality excluded, excluded because the paired counterpart failed, or pair-eligible but unselected. Participant-level accounting is reported in Supplementary Tables S1 and S2, whereas individual behavioral-exclusion counts are provided in Supplementary Table S4. For matched-probe trials, behavioral eligibility required that all applicable criteria be satisfied: VSA trials required *E* ≥ 5, *I* ≤ 3, and *A* ≥ 5, whereas VIDC trials required *I* ≥ 5, *E* ≤ 3, and *A* ≥ 5. In addition, the trial-level mean RT was required to be no greater than *μ*_cal_ + 2*σ*_cal_, within-trial RTV was required to be no greater than 2*σ*_cal_, and no omission was permitted among the four probes. For no-probe trials, only the corresponding post-trial rating criteria were applied. Failure to satisfy any applicable criterion resulted in behavioral exclusion [38,39,40,41,42]. The predefined behavioral criteria and participant-specific thresholds are reported in Supplementary Table S4. Candidate-trial schedules and participant-level trial and sample accounting are provided in Supplementary Tables S1 and S2.

### 3.2. Data Collection and Preprocessing

EEG data were collected from 16 healthy participants (8 females, 8 males; age: 18–23 years, mean 21.4 ± 1.3). All participants were right-handed, with normal or corrected-to-normal vision, and reported no history of neurological or psychiatric disorders. Prior to the experiment, participants were instructed to avoid substances affecting the central nervous system. The study was conducted in accordance with the Declaration of Helsinki and approved by the institutional review board (IRB) of our university. Written informed consent was obtained from all participants.

EEG signals were recorded from the prefrontal channels Fp1 and Fp2 using a Cyton + Daisy EEG acquisition system (OpenBCI, Inc., Brooklyn, NY, USA), with electrode placement according to the international 10–20 system [43], bilateral-earlobe referencing, a sampling rate of 250 Hz, and electrode impedance maintained below 5 kΩ. During data acquisition, participants were instructed to minimize unnecessary eye movements and blinking. All recordings underwent visual inspection to exclude segments exhibiting obvious residual ocular, motion, or high-frequency muscle artifacts. Before filtering and segmentation, the raw continuous recordings from Fp1 and Fp2 were independently inspected by two researchers, and any disagreements were resolved by consensus. Ocular artifacts were identified as prominent slow deflections or stereotyped blink-related transients; motion- or electrode-related artifacts as abrupt baseline shifts, signal discontinuities, or clipping; and muscle artifacts as irregular high-frequency bursts distinguishable from the background EEG. No numerical amplitude threshold was applied; identification was based on waveform morphology, temporal pattern, and channel distribution. Intervals meeting any of these criteria in either channel were marked as contaminated, and all 3 s segments overlapping these intervals were excluded. Raw EEG signals were subsequently filtered using a 5–40 Hz band-pass filter to suppress low-frequency drift, attenuate a substantial proportion of slow ocular activity, and remove high-frequency noise, while a 50 Hz notch filter was applied to remove power-line interference.

Each 72 s trial was initially divided into 24 fixed, non-overlapping 3 s candidate windows. For matched-probe trials, EEG data from probe onset to 3 s after probe onset were excluded. Because probe onsets were specified at integer-second resolution, a probe-related exclusion interval overlapped one fixed candidate window when the onset coincided with a 3 s window boundary and two candidate windows otherwise. As the four probe intervals were separated by at least 10 s, the exclusion intervals did not overlap. Consequently, four to eight candidate windows were removed, leaving 16–20 candidate windows before artifact rejection. No lower bound greater than 16 was imposed; a trial could therefore contain exactly 16 remaining windows. The corresponding virtual-probe intervals were excluded from no-probe trials to maintain comparable temporal sampling.

A trial was eligible only when at least 16 artifact-free candidate windows remained after probe-related or virtual-probe-related exclusion and artifact inspection. If more than 16 eligible windows remained, 16 windows were randomly selected using a fixed random seed of 42; if exactly 16 remained, all 16 were retained. Trials containing fewer than 16 artifact-free windows were excluded in their entirety. Trial eligibility was assessed individually, followed by pair-level retention of VSA and VIDC trials sharing the same Pair ID. Exactly five eligible pairs were selected in each probe condition. When fewer than five eligible pairs were available among the eight initial pairs, additional paired trials were collected; three participants required nine additional paired sets, corresponding to 18 individual trials.

This procedure yielded 80 samples for each attentional-state × probe-condition combination per participant (5 trials × 16 samples), corresponding to 160 samples per participant in each probe condition. Across the 16 participants, each experimental combination contained 1280 samples, while the matched-probe and no-probe datasets each contained 2560 samples. Identical sample counts were retained in all four within-condition and cross-condition training–test configurations.

To assess the sensitivity of PDC-Net to residual ocular and myogenic contamination, an additional combined artifact-robustness analysis was conducted on the primary matched-probe dataset. Following the initial visual inspection, the remaining 5–40 Hz candidate windows were subjected to additional label-independent screening based on peak-to-peak amplitude, maximum absolute first derivative, kurtosis, and relative 30–40 Hz power. For each participant and channel, a window was excluded when any measure exceeded the participant- and channel-specific median plus six median absolute deviations; a window was rejected when either Fp1 or Fp2 met an exclusion criterion. The retained data were then reprocessed using a restricted 5–30 Hz passband. The actual-probe exclusion procedure, trial-eligibility criteria, LOSO partitions, normalization strategy, model architecture, and training hyperparameters remained unchanged. When a retained trial no longer provided 16 eligible windows, an eligible backup trial from the same participant, attentional state, and probe condition was used. Consequently, the combined analysis retained 80 VSA and 80 VIDC samples per participant. Artifact screening and sample selection were completed without reference to attentional-state labels or classification results.

### 3.3. Baseline Models

Ten representative baselines were included, covering traditional machine-learning, convolutional, and EEG-oriented hybrid architectures. All models were evaluated using the same participant-level LOSO partitions; their architecture-specific settings and optimization procedures are described below and in Supplementary Table S5.

The traditional machine-learning baselines comprised an SVM, for which radial-basis-function and polynomial kernels were considered during tuning, Random Forest (RF) [44], and Extreme Gradient Boosting (XGBoost) [45]. The SVM and RF models were implemented using scikit-learn version 1.3.2, whereas the XGBoost model was implemented using the XGBoost Python package version 1.7.6. Each preprocessed 3 s Fp1/Fp2 EEG segment was represented by a handcrafted feature vector. Welch’s method was used to estimate the power spectral density of the 5–40 Hz signals. Absolute power was computed for theta (5–8 Hz), alpha (8–13 Hz), beta (13–30 Hz), and gamma (30–40 Hz), and spectral entropy was calculated from the normalized 5–40 Hz power spectral energy [46]. The mean, standard deviation, skewness, kurtosis, and three Hjorth parameters were also extracted, yielding 12 features per channel and 24 features per segment. Z-score normalization parameters were estimated from the training partition of each LOSO fold and applied to the corresponding validation and test partitions.

The deep-learning baselines included EEGNet [11], DeepConvNet and ShallowConvNet [12], TSception [47], EEGConformer [48], Convolutional Transformer Network (CTNet) [49], and EEGNeX [50]. All models were systematically validated using the same LOSO cross-validation framework to ensure a fair and comprehensive performance comparison. Their architecture-specific configurations were adapted to the common two-channel input containing 750 time points and are reported in Supplementary Table S5. The models were implemented using Braindecode version 1.6.1, an open-source framework [51]. Parameters intrinsic to each architecture, including convolutional filter numbers, temporal kernel sizes, pooling settings, Transformer depth, number of attention heads, and dropout, were configured separately rather than being assumed to be identical across models. These architecture-specific parameters were fixed during optimization, whereas the same learning-rate and weight-decay search space was evaluated for every deep-learning model. The principal fixed settings and complete hyperparameter-search spaces for all compared models are reported in Supplementary Table S5.

### 3.4. Experimental Workflow and Validation Paradigm

All experiments were implemented using PyTorch 2.1.1 with Python 3.10.14 on a system equipped with an Intel Xeon Platinum 8255C CPU (12 vCPUs; Intel Corporation, Santa Clara, CA, USA) and an NVIDIA RTX 2080 Ti GPU (11 GB memory; NVIDIA Corporation, Santa Clara, CA, USA). All deep-learning models used cross-entropy loss, Adam optimization, a batch size of 32, a maximum of 100 epochs, and gradient clipping with a maximum norm of 1.0. For each deep-learning model, the initial learning rate was selected from {1 × 10^−4^, 5 × 10^−4^, 1 × 10^−3^}, and weight decay was selected from {0, 1 × 10^−4^, 1 × 10^−3^}, yielding the same nine candidate optimization configurations per model. Architecture-specific parameters and dropout values were fixed as reported in Supplementary Table S5. Crucially, to avoid data leakage during subject-independent evaluation, normalization parameters were computed exclusively from the active training partition in each fold and were subsequently applied unchanged to the corresponding validation and test participants. Under the LOSO framework, all data from one participant were reserved exclusively as the held-out test set in each fold. For each fold, the IDs of the remaining 15 participants were shuffled using a fold-specific random seed defined as 42 plus the zero-based fold index. The first two participants in the shuffled sequence were assigned in their entirety to the validation set, while the other 13 participants constituted the training set. Thus, the division was performed at the participant level rather than the EEG-segment level, and the training, validation, and test sets were mutually exclusive at both the participant and segment levels.

To provide comparable optimization effort while preserving the model-specific characteristics of each architecture, the subject-level partitions, input EEG samples, preprocessing procedure, validation criterion, and maximum training duration were held consistent across models where applicable. Architecture-specific parameters, including convolutional filter numbers, temporal kernel sizes, pooling settings, Transformer depth, number of attention heads, and dropout, were configured separately and fixed as reported in Supplementary Table S5. For the traditional machine-learning models, all configurations in the corresponding model-specific grids were trained using the 13 training participants and evaluated on the two fixed validation participants. The SVM, RF, and XGBoost searches comprised 32, 36, and 54 candidate configurations, respectively. For every deep-learning model, including PDC-Net, the same 3 × 3 grid comprising three initial learning rates and three weight-decay values was evaluated, yielding nine candidate optimization configurations per model. Hyperparameters and model checkpoints were selected exclusively according to validation performance within the corresponding outer LOSO fold. No data from the held-out test participant were used for normalization, hyperparameter selection, early stopping, or checkpoint selection. The principal fixed parameters, complete search spaces, and shared optimization controls are reported in Supplementary Table S5.

For inferential comparisons between PDC-Net and the baseline models, the accuracy obtained for each of the 16 held-out participants was treated as one paired observation. For each baseline, the participant-level accuracy difference was calculated as PDC-Net minus the corresponding baseline. Two-sided Wilcoxon signed-rank tests were performed because of the limited number of participants and the absence of an assumption of normally distributed paired differences [52]. The resulting *p*-values were adjusted across all ten baseline comparisons using the Holm procedure. Ninety-five percent confidence intervals for the mean paired accuracy differences were estimated using 10,000 participant-level paired bootstrap resamples. Effect sizes were reported as matched-pairs rank-biserial correlations (*r*_rb_), with positive values indicating higher accuracy for PDC-Net. Statistical comparisons involving the ablation variants were conducted separately and were used only to evaluate the contributions of the corresponding architectural components. The fold-specific participant partitions were generated once and reused unchanged for every compared model. Each deep-learning configuration was trained once per outer fold using the same training random seed of 42; neither the validation partition nor the training seed was repeatedly resampled, and the reported results were not averaged across multiple random-seed runs.

## 4. Results

### 4.1. Cross-Subject Decoding Experiment

**Figure 4:**
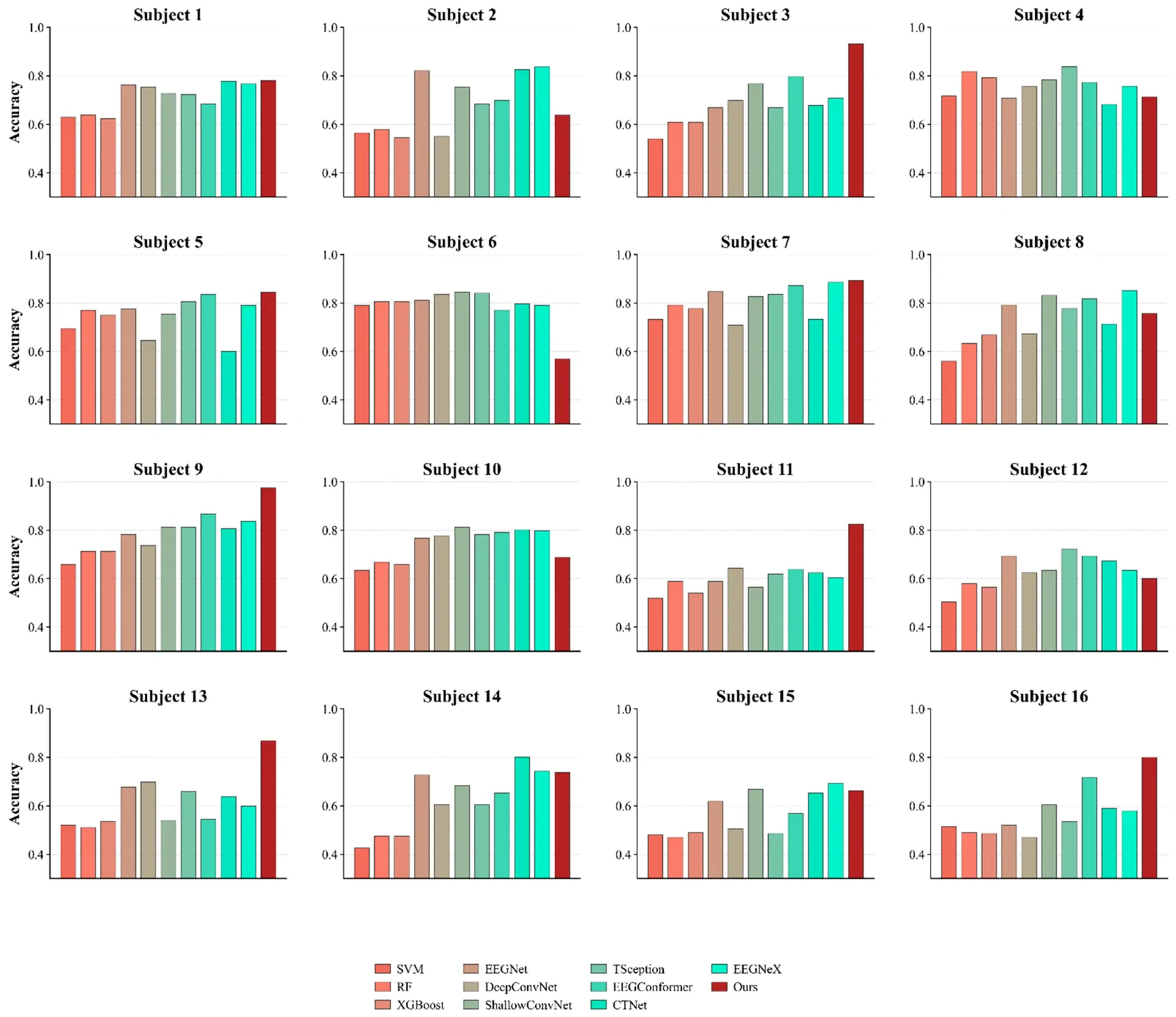
Subject-wise classification accuracy across all compared models and the proposed PDC-Net for each participant (Subjects 1–16) under LOSO cross-validation.

Under the LOSO protocol, PDC-Net achieved the highest mean accuracy among the evaluated models, with an accuracy of 76.76 ± 11.90% and an AUC of 0.855 ± 0.110. Although PDC-Net achieved the highest mean accuracy, it did not achieve the highest mean AUC. EEGNeX and CTNet obtained mean AUC values of 0.874 ± 0.091 and 0.860 ± 0.106, respectively, compared with 0.855 ± 0.110 for PDC-Net. Participant-level paired comparisons with the seven deep-learning baselines showed that PDC-Net achieved significantly higher accuracy than EEGNet, DeepConvNet, ShallowConvNet, TSception, and EEGConformer after Holm correction, whereas the differences relative to CTNet and EEGNeX did not reach statistical significance. Precision (Pre), recall (Rec), and F1-score are also reported for the VSA (+) and VIDC (−) classes, together with their macro-averages (avg), to provide a balanced evaluation. The individual-participant results are presented in Figure 4. Compared with EEGNeX, which achieved the second-highest mean accuracy, PDC-Net showed an absolute mean accuracy advantage of 2.58 percentage points.

The cross-category comparison in Table 2 reveals a clear performance hierarchy under the evaluated cross-subject LOSO setting. Specifically, the traditional machine-learning models yielded lower mean accuracies than the evaluated deep-learning architectures, suggesting that the selected handcrafted features provided comparatively limited cross-subject discriminability in the present dataset. The highest-accuracy CNN-based baseline, EEGNeX, obtains an average accuracy of 74.18%, which is 2.58% lower than that of the proposed PDC-Net. The best CNN-Transformer hybrid model, EEGConformer, achieves an accuracy of 72.34% and still exhibits a 4.42-percentage-point difference in mean accuracy relative to PDC-Net. These results suggest that increasing network depth or introducing self-attention alone may not be sufficient to effectively model the cross-subject dynamics of two-channel prefrontal EEG. The participant-level paired comparisons in Table 2b further quantify the accuracy differences between PDC-Net and the individual baseline models. In the module-removal analysis reported in Section 4.3, removing CGMI reduced the mean accuracy from 76.76 ± 11.90% to 66.21 ± 12.00%, corresponding to a decrease of 10.55 percentage points (*p* = 0.0016). This result indicates that the performance of the implemented PDC-Net architecture was sensitive to the presence of CGMI. However, because removal of CGMI also changed the parameter count, model capacity, and computational pathway, this comparison does not isolate the mechanism-specific contribution of CGMI independently of these architectural changes. Moreover, PDC-Net was trained using a standard classification objective without an explicit subject-invariance or inter-subject distribution-alignment objective. Therefore, the results support the contribution of CGMI to decoding performance within the implemented architecture but do not demonstrate that CGMI eliminates or explicitly aligns inter-subject distribution shifts.

To further characterize the computational properties of the evaluated models under the offline experimental setting, their computational costs were compared in terms of the number of trainable parameters, multiply–accumulate operations (MACs), floating-point operations (FLOPs), and single-sample GPU inference latency, as shown in Table 3. The number of trainable parameters was used as an architecture-level indicator of model size. All models were evaluated under a consistent setting using a 3 s, two-channel EEG segment containing 750 time points and a batch size of 1. Inference latency was measured in FP32 mode on the same NVIDIA RTX 2080 Ti GPU after model warm-up and is reported as the mean ± standard deviation over repeated forward passes. As shown in Table 3, EEGNet achieved the lowest parameter count and theoretical computational cost, whereas ShallowConvNet exhibited the lowest measured GPU latency. PDC-Net required fewer MACs and FLOPs than DeepConvNet, ShallowConvNet, EEGConformer, CTNet, and EEGNeX, although it did not achieve the lowest parameter count or measured latency. Its measured latency was lower than those of all evaluated deep-learning models except EEGNet and ShallowConvNet. Accordingly, PDC-Net did not minimize every individual complexity metric but achieved the highest mean decoding accuracy among the compared models while maintaining moderate operation counts and GPU inference latency. Taken together, these results indicate a favorable balance between cross-subject decoding performance and computational cost under the evaluated offline GPU setting. They should not be interpreted as measurements of CPU/ARM latency, peak runtime memory, energy consumption, or end-to-end streaming performance on wearable hardware.

An additional combined artifact-robustness analysis was performed to evaluate the sensitivity of the primary result to more conservative artifact screening and removal of the 30–40 Hz frequency range. Under this combined pipeline, PDC-Net achieved an accuracy of 75.47 ± 11.92%, an F1-score of 0.725 ± 0.125, an AUC of 0.822 ± 0.111, and a Kappa value of 0.509 ± 0.238 (Supplementary Table S3). Relative to the primary analysis, the mean participant-level accuracy difference was −1.29 percentage points (95% CI: [−2.73, 0.12]; Wilcoxon signed-rank statistic = 32.0, two-sided *p* = 0.112, *r*_rb_ = −0.467). Thus, no statistically significant difference in participant-level accuracy was detected between the primary and combined-robustness analyses. The combined-robustness accuracy also remained significantly above the nominal 50% chance level (one-sided Wilcoxon signed-rank statistic = 136.0, *p* < 0.001). Artifact-burden accounting based on the recorded artifact logs showed 9.13 ± 2.53 rejected windows for VSA-MP and 10.13 ± 2.09 for VIDC-MP, with no statistically significant condition difference detected (Wilcoxon signed-rank statistic = 25.0, *p* = 0.081). These findings indicate that the principal decoding pattern persisted under the combined conservative preprocessing perturbation and that no marked state-dependent difference in logged artifact burden was detected.

### 4.2. Probe-Control and Cross-Condition Generalization Analyses

To examine whether the decoding performance depended on the presence or temporal structure of the probes, three additional subject-independent analyses were conducted using the no-probe data. These analyses comprised: (1) training and testing within the no-probe condition (No-probe → No-probe); (2) training on the matched-probe condition and testing on the no-probe condition (Matched-probe → No-probe); and (3) training on the no-probe condition and testing on the matched-probe condition (No-probe → Matched-probe). The primary Matched-probe → Matched-probe result reported in Section 4.1 was also included in Table 4 as a reference.

For comparability between probe conditions, each NP trial was assigned a virtual probe schedule corresponding to one of the MP schedules. Candidate 3 s windows overlapping the intervals following these virtual probe onsets were excluded only for temporal-matching purposes, although no actual probes were presented. From each retained trial, 16 artifact-free windows were selected using the same prespecified procedure in both probe conditions. Consequently, the MP and NP datasets each contained 80 VSA and 80 VIDC samples per participant, yielding 160 samples per participant with equivalent class sizes and matched within-trial temporal sampling. This procedure prevented differences in sample number or temporal coverage from contributing systematically to the comparisons between probe conditions.

All four training–testing configurations followed the same subject-level LOSO framework. In each outer fold, the same participant was reserved as the entirely unseen test subject, and the remaining participants were assigned to the same training and validation partitions used in the primary experiment. For the two cross-condition analyses, model fitting, early stopping, and normalization were performed exclusively using the source-condition data from the training and validation participants. The normalization parameters calculated from the source-condition training data were then applied directly to the held-out participant’s target-condition data. No target-condition data from the held-out participant were used for model fitting, hyperparameter selection, normalization, or fine-tuning. The architecture and training hyperparameters of PDC-Net were unchanged across the four configurations.

As shown in Table 4, PDC-Net achieved an accuracy of 74.38 ± 10.06% in the NP → NP analysis, representing a decrease of 2.38 percentage points relative to the primary MP → MP result. The 95% confidence interval of the participant-level paired difference was [−0.34, 5.38] percentage points, and no statistically significant difference in accuracy was detected between the MP → MP and NP → NP configurations after Holm correction (adjusted *p* = 0.183). Importantly, the NP → NP accuracy remained descriptively well above the nominal chance level of 50% despite the absence of actual probe presentation, probe-related motor responses, and repeated probe-related interruptions. When trained on MP data and evaluated on NP data, PDC-Net achieved an accuracy of 71.33 ± 8.38%, corresponding to a decrease of 5.43 percentage points relative to MP → MP (95% CI: [1.47, 8.01]; adjusted *p* = 0.019). In the reverse NP → MP direction, the model achieved an accuracy of 70.27 ± 9.93%, corresponding to a decrease of 6.49 percentage points (95% CI: [3.46, 9.28]; adjusted *p* = 0.012). Thus, both cross-condition configurations showed significantly lower accuracy than the MP → MP reference condition, while their mean accuracies remained descriptively above the nominal chance level.

Taken together, the comparable MP → MP and NP → NP accuracies, together with the bidirectional cross-condition results, provide evidence that VSA/VIDC decoding was not predominantly dependent on differences in probe presentation or temporal structure. The lower bidirectional cross-condition accuracies suggest that the learned representations were only partially transferable between the MP and NP conditions. These findings reduce, but do not eliminate, the possibility that the primary decoding performance was influenced by the presence or temporal structure of the probes. Because participants were aware that probes could occur during the experiment, a residual general anticipatory task set cannot be completely excluded.

### 4.3. Module-Removal Ablation Analysis

**Figure 5:**
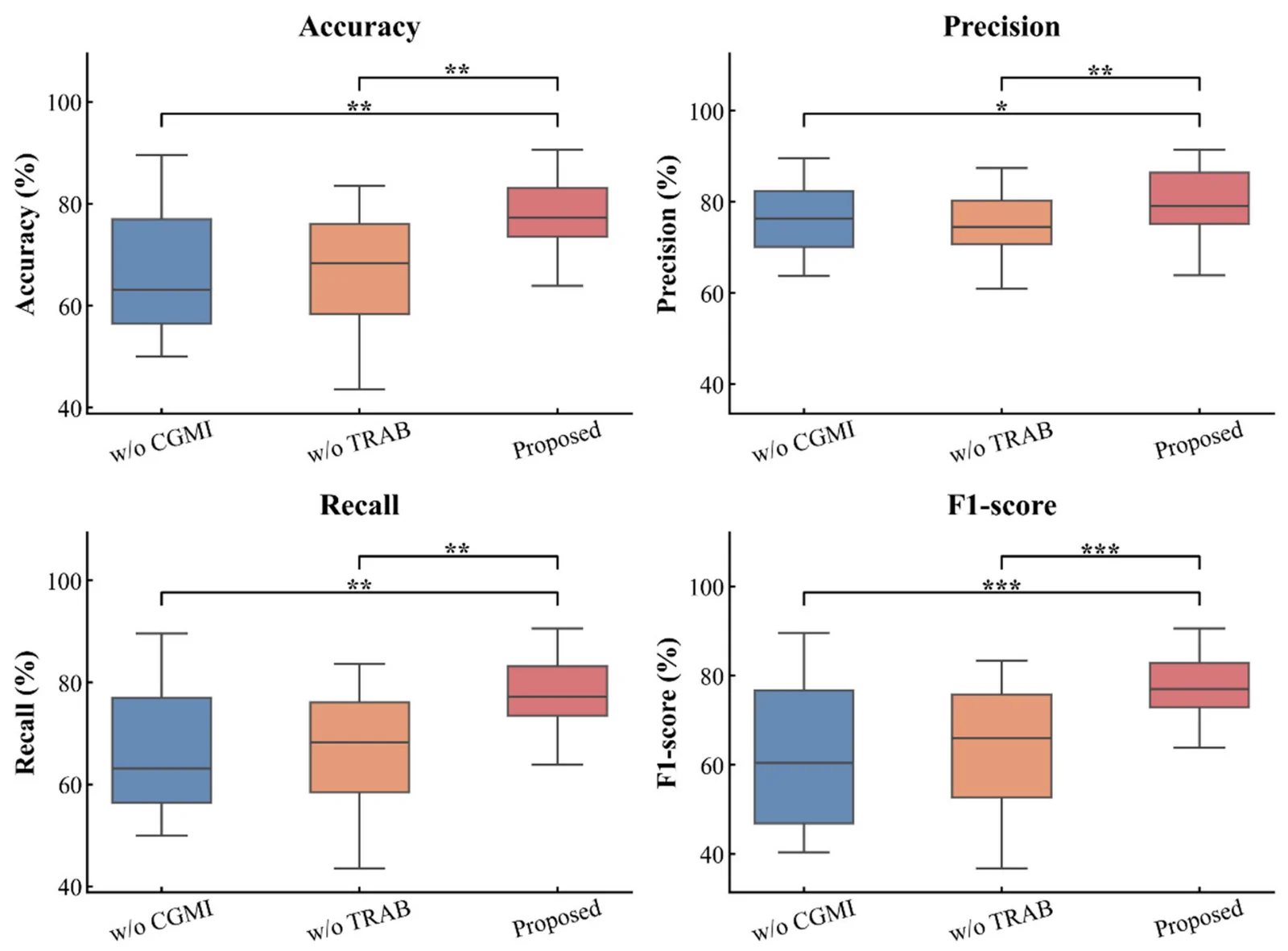
Box plots of performance metrics from the module-removal ablation analysis, comparing the complete PDC-Net with variants w/o TRAB and w/o CGMI. The reduced variants were not parameter-matched to the complete model. * *p* < 0.05, ** *p* < 0.01, and *** *p* < 0.001, based on paired Wilcoxon signed-rank tests.

A module-removal ablation analysis was conducted under the same LOSO cross-validation protocol to quantify the performance changes associated with excluding TRAB or CGMI from the complete PDC-Net architecture. The complete model was compared with two variants in which either TRAB or CGMI was removed, while the remaining experimental settings were kept unchanged. These comparisons were not parameter-matched because removing either module also reduced model capacity and altered the corresponding computational pathway. Participant-level differences between the complete and reduced models were assessed using paired Wilcoxon signed-rank tests [52], and the resulting performance distributions are shown in Figure 5.

Removing either module was associated with lower decoding performance. For accuracy, comparisons of the complete model with the variants without CGMI and without TRAB yielded *p* = 0.0016 and *p* = 0.0024, respectively. The corresponding *p*-values were 0.0214 and 0.0021 for precision, 0.0016 and 0.0060 for recall, and 0.0002 and 0.0004 for F1-score. Specifically, removing TRAB reduced the accuracy from 76.76 ± 11.90% to 66.72 ± 11.59%, corresponding to a decrease of 10.04 percentage points. Removing CGMI reduced the accuracy to 66.21 ± 12.00%, corresponding to a decrease of 10.55 percentage points. These results indicate that the performance of the complete PDC-Net architecture was sensitive to the presence of both modules. Because the reduced variants were not parameter-matched, the observed differences should not be interpreted as isolating the mechanism-specific benefit of either module independently of model capacity and architectural structure.

The stage-wise t-SNE projections presented in Section 4.4 provide a descriptive visualization of changes in the projected feature distributions across the processing stages [53,54]; they are not used as parameter-matched or causal evidence for the effect of either module.

### 4.4. Visualization Analysis

**Figure 6:**
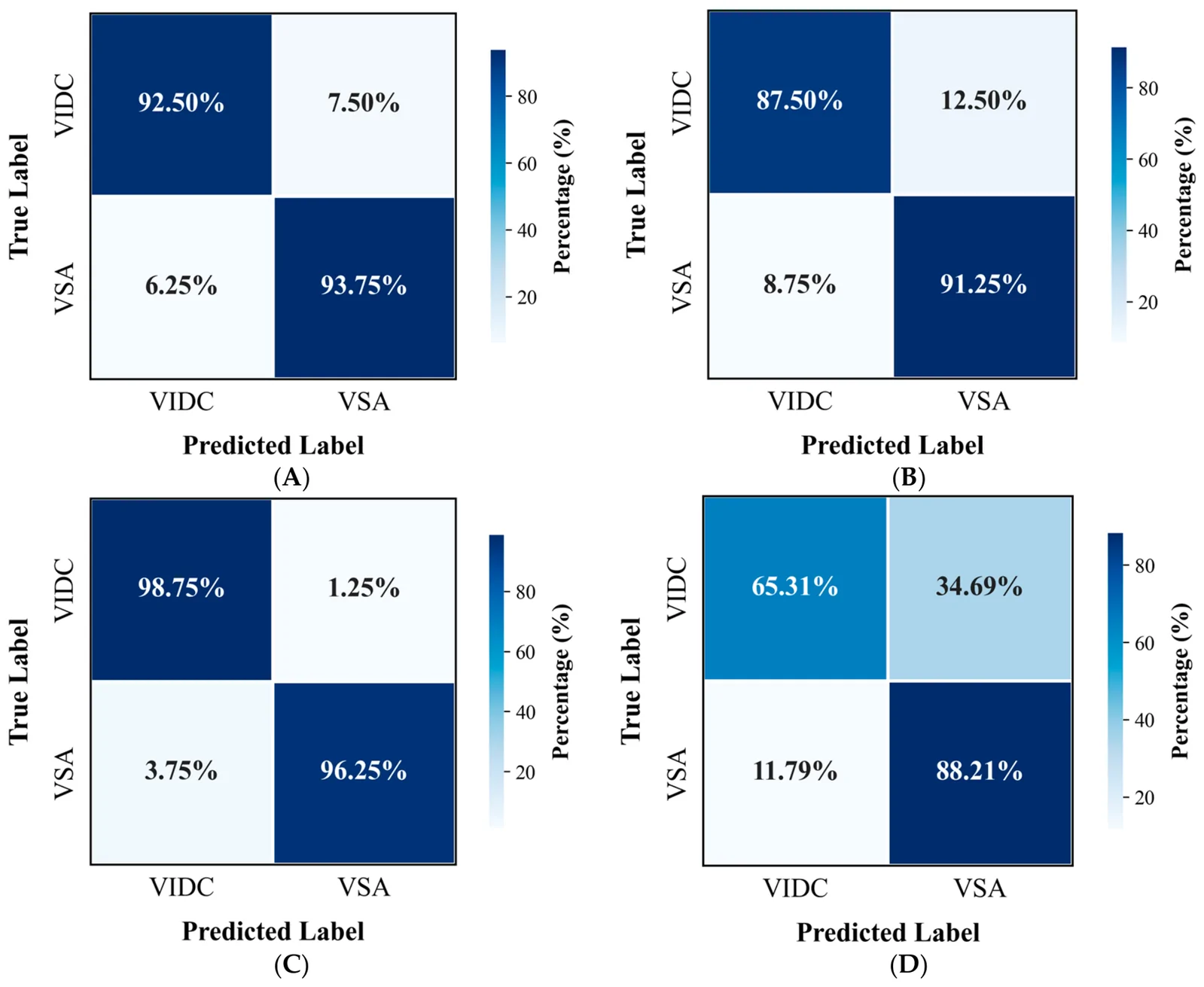
Confusion matrices of the proposed PDC-Net for two-class VSA/VIDC state classification. (**A**–**C**) Normalized confusion matrices for the three best-performing individual subjects: Subject 3 (93.13%), Subject 7 (89.38%), and Subject 9 (97.50%); (**D**) average confusion matrix across all 16 participants. Label 0 denotes the VIDC state, and label 1 denotes the VSA state. Each cell reports the percentage of samples in the corresponding true-label row assigned to each predicted class; therefore, the values in each row sum to 100%.

The normalized confusion matrices in Figure 6 provide a descriptive summary of class-specific prediction patterns for the three top-performing participants and the average across all 16 participants. The matrices exhibit dominant diagonal distributions. At the group level, the mean class-specific accuracies were 88.21% for VSA and 65.31% for VIDC, indicating that VSA was classified more accurately than VIDC. These results describe the distribution of correct and incorrect predictions but do not independently establish neurophysiological plausibility, biological consistency, labeling validity, or practical monitoring performance.

**Figure 7:**
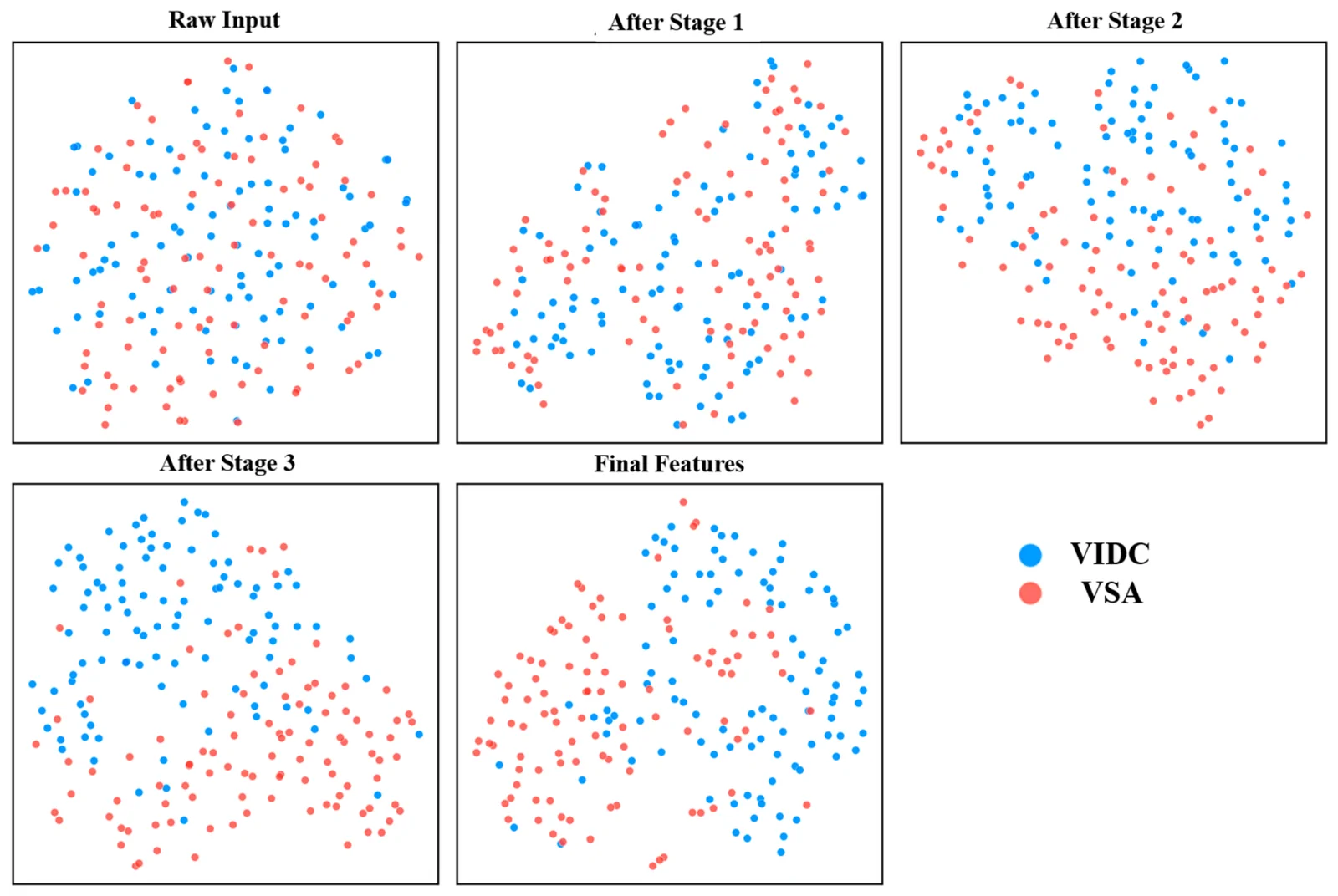
Qualitative t-SNE projections of EEG feature distributions across different processing stages of PDC-Net. The five panels correspond to the raw input, the outputs of Stages 1–3, and the final feature representation. Each panel contains the same stratified subset of 200 training samples, comprising 100 VIDC and 100 VSA samples from Subjects 2–9, 11–14, and 16. A separate t-SNE projection was fitted at each stage using a perplexity of 30, a learning rate of 200, 1000 optimization iterations, PCA initialization, and a random seed of 42. Because the projections were fitted independently, their coordinate systems are not directly comparable across panels.

The t-SNE visualization qualitatively illustrates changes in the feature representations across the PDC-Net processing pipeline. The visualization was generated using a stratified subset of 200 training samples, comprising 100 VIDC and 100 VSA samples, from Subjects 2–9, 11–14, and 16 in the LOSO fold in which Subject 1 was held out for testing and Subjects 10 and 15 were used for validation. Subjects 2–9 and 11 each contributed eight samples per class, whereas Subjects 12–14 and 16 each contributed seven samples per class. No samples from the validation or held-out test participants were included. The trained model from this LOSO fold was used to extract the raw input representation, the outputs of Stages 1–3, and the final feature representation. For each processing stage, a separate t-SNE projection was fitted to the same 200 samples using a perplexity of 30, a learning rate of 200, 1000 optimization iterations, PCA initialization, and a fixed random seed of 42. The sample selection and t-SNE analysis were performed only for post hoc visualization and did not affect model training, hyperparameter selection, or performance evaluation. As shown in Figure 7, the raw-input projection exhibits substantial overlap between the two classes. Class-related clustering patterns become more discernible in the later processing stages, particularly after Stage 3 and in the final feature representation, although some overlap remains. Because t-SNE is nonlinear and stochastic, it can distort global distances, and because it was fitted independently at each stage, the projections are interpreted only as qualitative views of the selected training samples [53,54]. They do not provide quantitative evidence of class separability, cross-subject alignment, subject invariance, neurophysiological validity, or a causal effect of CGMI.

## 5. Discussion

Cross-subject decoding of visual attentional states remains challenging because of inter-subject EEG variability. This challenge is compounded by the limited spatial information available in reduced-channel recording configurations [55]. The present study evaluated PDC-Net, a dual-branch architecture operating on prefrontal Fp1/Fp2 EEG signals [5,43]. Within this architecture, TRAB performs local temporal feature transformation and feature-channel recalibration, whereas CGMI enables gated cross-branch interaction [23,28]. Under the evaluated LOSO protocol, PDC-Net achieved a mean accuracy of 76.76 ± 11.90%, representing the highest mean accuracy among the compared models. These results support the feasibility of VSA/VIDC decoding using two EEG acquisition channels under the present controlled experimental conditions [22], but they do not independently establish the neurophysiological specificity or biological validity of the learned representations.

Participant-level comparisons showed significant accuracy differences in favor of PDC-Net over eight baselines, whereas the differences relative to CTNet and EEGNeX were not significant after Holm correction. The module-removal results indicate that performance was sensitive to both TRAB and CGMI; however, because the variants were not parameter-matched, these comparisons cannot isolate mechanism-specific effects. Similarly, t-SNE was used only as a qualitative visualization and does not establish subject-invariant alignment or neurophysiological validity [54].

CGMI is one of several possible bilateral fusion strategies. Unlike direct concatenation or subtraction, it retains separate branch representations and uses their difference only to generate input-dependent gates. Compared with static or pointwise MLP gating, its gating pathway incorporates selective state-space sequence modeling; unlike cross-attention, it retains linear sequence complexity [23]. However, the present study did not compare CGMI with these alternatives under matched parameter and computational budgets. Its relative performance against concatenation, subtraction, static gating, MLP gating, cross-attention, and plain Mamba fusion, therefore, remains to be evaluated.

TRAB combines pointwise transformation, fixed-kernel depthwise convolution, and feature-channel recalibration; stacking these blocks enlarges the effective receptive field without parallel multi-kernel or dilated convolutions. CGMI complements this pathway through gated cross-branch interaction. The module-removal results support retaining both modules within the implemented architecture but do not isolate their effects from changes in model capacity. PDC-Net also uses selective state-space modeling with linear sequence complexity [23]. Nevertheless, the empirical comparison in Table 3 shows that linear sequence complexity does not imply the lowest overall model cost. EEGNet achieved the lowest parameter count and MACs, while ShallowConvNet [12] achieved the lowest measured latency. PDC-Net required 0.939 M parameters, 3.516 M MACs, and 7.109 M FLOPs but maintained a sub-millisecond inference latency of 0.460 ± 0.089 ms. With respect to operation counts, PDC-Net required fewer MACs and FLOPs than DeepConvNet and ShallowConvNet [12], EEGConformer [48], CTNet [49], and EEGNeX [50], although its parameter count exceeded those of several baselines. Accordingly, PDC-Net is interpreted as providing a favorable performance–computational-cost trade-off under the evaluated offline GPU setting rather than a universal computational advantage.

## 6. Conclusions

This study presents PDC-Net for cross-subject VSA/VIDC decoding from dual-channel Fp1/Fp2 EEG. Under offline LOSO evaluation, PDC-Net achieved the highest mean accuracy among the compared models and a favorable balance between decoding performance and computational cost. Its hierarchical temporal processing and gated cross-branch interaction provide a practical framework for reduced-channel EEG decoding. These findings support further external, cross-session, online, and hardware-specific evaluation.

Several limitations should be acknowledged. First, the study included only 16 healthy young adults within a narrow age range, which may limit generalizability to broader age groups and clinical populations. In addition, each outer LOSO fold used one prespecified two-participant validation set rather than repeated inner resampling. Although the same fold-specific partitions were used for all models, model selection may remain sensitive to validation-set composition. Larger studies could address this variability using repeated nested participant-level validation.

Second, the findings were obtained from controlled, offline, single-session recordings without independent external, cross-session, online, edge-device, or clinical validation. Accordingly, the reported LOSO accuracy and GPU latency characterize only the evaluated offline setting. Continuous real-world recordings may introduce motion, variable electrode contact, environmental distractions, and temporal distribution shifts. These concerns are particularly relevant to Fp1/Fp2 because dedicated EOG and facial EMG channels were not recorded. Although stricter artifact screening and a restricted 5–30 Hz passband produced a comparable decoding pattern, this sensitivity analysis cannot exclude residual ocular or myogenic contributions. Future studies should incorporate synchronized vertical and horizontal EOG and, where feasible, facial EMG recordings for independent artifact quantification and removal [56,57]. The present paradigm also addresses only coarse-grained sustained attentional states and does not characterize transient attentional shifts or finer fluctuations in engagement.

Third, PDC-Net does not include an explicit subject-invariance objective or inter-subject distribution-alignment constraint, and the present study did not quantify subject-identity information or inter-subject feature-distribution distances. The contribution of CGMI should therefore be interpreted as improved decoding performance within the implemented architecture rather than as evidence that it eliminates inter-subject distribution shifts. Future studies could incorporate subject-identity classification, feature-distance measures, and explicit domain-alignment strategies to evaluate whether the learned representations reduce subject-specific information [16,58].

Finally, the module-removal variants were not matched to the complete model in parameter count or computational structure. The observed reductions, therefore, show that PDC-Net was sensitive to the removal of TRAB and CGMI but do not isolate their mechanism-specific effects from the accompanying changes in model capacity. Parameter-matched residual-convolution and alternative-fusion controls should be considered in future work.

Future work will therefore focus on validating the proposed framework in larger and more demographically diverse cohorts, including participants from different age groups and, where appropriate, relevant clinical populations. Independent cross-session and multicenter evaluations will be conducted to examine robustness to temporal and population-level distribution shifts. In parallel, real-time artifact detection, signal-quality monitoring, and adaptive learning strategies should be integrated to improve performance during continuous wearable recording. Prospective studies in representative use scenarios, such as neurofeedback, attention training, and longitudinal cognitive-state monitoring, will also be necessary to determine whether the present offline decoding performance translates into reliable real-time benefits. Finally, further model compression and on-device benchmarking will be considered to evaluate memory consumption, energy requirements, and end-to-end latency on actual edge hardware rather than relying solely on GPU-based inference measurements. These extensions would provide a more comprehensive basis for assessing the practical utility of PDC-Net in wearable brain–computer interface applications.

## Figures and Tables

**Table 1:** Stage-wise architecture and key configurations of PDC-Net for a 3 s dual-channel EEG input. *B*, *L*, and *D* denote batch size, sequence length, and feature dimension, respectively; *k*, *s*, *d*, and *p* denote kernel size, stride, dilation, and padding.

Stage	Main Operation	Key Configuration	Output Dimension
Input	Fp1/Fp2 EEG	*T* = 750, sampled at 250 Hz	(*B*, 2, 750)
Embedding	Independent branch Conv1D	1 → 32, *k* = 3, *s* = 1, *p* = 1	2 × (*B*, 32, 750)
Stage 1	TRAB + CGMI	*D*_1_ = 32; TRAB: expansion = 2, *k* = 15, *s* = 1, *d* = 1, *p* = 7; Mamba: *d*_state_ = 16, *d*_conv_ = 4, expand = 2, *dt*_rank_ = 2	2 × (*B*, 750, 32)
Downsampling 1	Conv1D	32 → 64, *k* = 3, *s* = 2, *p* = 1	2 × (*B*, 64, 375)
Stage 2	TRAB + CGMI	*D*_2_ = 64; TRAB: expansion = 2, *k* = 15, *s* = 1, *d* = 1, *p* = 7; Mamba: *d*_state_ = 16, *d*_conv_ = 4, expand = 2, *dt*_rank_ = 4	2 × (*B*, 375, 64)
Downsampling 2	Conv1D	64 → 128, *k* = 3, *s* = 2, *p* = 1	2 × (*B*, 128, 188)
Stage 3	TRAB + CGMI	*D*_3_ = 128; TRAB: expansion = 2, *k* = 15, *s* = 1, *d* = 1, *p* = 7; Mamba: *d*_state_ = 16, *d*_conv_ = 4, expand = 2, *dt*_rank_ = 8	2 × (*B*, 188, 128)
Alignment and aggregation	Element-wise addition + Align + GAP	Align: (*B*, *L*, *D*) → (*B*, *D*, *L*)	(*B*, 128)
Classification head	Fully connected layers	128 → 64 → 2, dropout = 0.2	(*B*, 2)

**Table 2:** (**a**) Performance comparison in the cross-subject decoding experiment using LOSO cross-validation. Data are presented as mean ± SD. Acc, accuracy; Pre, precision; Rec, recall; F1, F1-score; AUC, area under the receiver operating characteristic curve; Kappa, Cohen’s kappa coefficient. The best and second-best mean performances for each metric are highlighted in bold and italics, respectively. (**b**) Participant-level paired statistical comparisons of accuracy between PDC-Net and each baseline model. ΔAcc denotes the participant-level mean paired accuracy difference, calculated as PDC-Net minus the corresponding baseline and expressed in percentage points. Confidence intervals were estimated using participant-level paired bootstrap resampling with 10,000 iterations. Two-sided Wilcoxon signed-rank tests were performed, followed by Holm correction across all baseline comparisons. Effect sizes are reported as matched-pairs rank-biserial correlations (*r*_rb_); positive values favor PDC-Net.

(**a**)												
**Model**	**Acc (%)**	**Pre**			**Rec**			**F1**			**AUC**	**Kappa**
		**+**	**−**	**Avg**	**+**	**−**	**Avg**	**+**	**−**	**Avg**		
SVM	59.26 ± 10.39	0.590 ± 0.100	0.600 ± 0.122	0.595 ± 0.108	0.528 ± 0.202	0.658 ± 0.071	0.593 ± 0.104	0.549 ± 0.155	0.622 ± 0.076	0.585 ± 0.110	0.630 ± 0.134	0.185 ± 0.208
RF	63.40 ± 11.86	0.637 ± 0.107	0.646 ± 0.153	0.641 ± 0.124	0.573 ± 0.231	0.694 ± 0.090	0.634 ± 0.119	0.591 ± 0.170	0.660 ± 0.091	0.625 ± 0.125	0.675 ± 0.148	0.268 ± 0.237
XGBoost	62.70 ± 11.44	0.625 ± 0.101	0.642 ± 0.148	0.634 ± 0.120	0.574 ± 0.228	0.680 ± 0.080	0.627 ± 0.115	0.587 ± 0.166	0.651 ± 0.084	0.619 ± 0.120	0.670 ± 0.148	0.254 ± 0.229
EEGNet	72.23 ± 9.07	0.725 ± 0.071	0.795 ± 0.169	0.759 ± 0.087	0.736 ± 0.287	0.709 ± 0.168	0.722 ± 0.091	0.694 ± 0.189	0.717 ± 0.076	0.705 ± 0.113	0.851 ± 0.115	0.445 ± 0.181
DeepConvNet	66.72 ± 10.01	0.621 ± 0.080	*0.832 ± 0.174*	0.726 ± 0.115	**0.899 ± 0.119**	0.437 ± 0.174	0.667 ± 0.100	0.730 ± 0.079	0.552 ± 0.173	0.641 ± 0.118	0.811 ± 0.156	0.334 ± 0.200
ShallowConvNet	72.54 ± 9.88	*0.741 ± 0.044*	0.786 ± 0.191	0.764 ± 0.095	0.697 ± 0.312	*0.754 ± 0.132*	0.725 ± 0.099	0.678 ± 0.198	*0.739 ± 0.048*	0.708 ± 0.118	0.843 ± 0.109	0.451 ± 0.198
TSception	71.21 ± 11.11	0.702 ± 0.082	0.751 ± 0.169	0.727 ± 0.118	0.712 ± 0.237	0.712 ± 0.079	0.712 ± 0.111	0.694 ± 0.155	0.719 ± 0.082	0.706 ± 0.115	0.797 ± 0.145	0.424 ± 0.222
EEGConformer	72.34 ± 8.65	0.718 ± 0.079	0.805 ± 0.151	0.762 ± 0.076	0.780 ± 0.235	0.667 ± 0.188	0.723 ± 0.086	0.720 ± 0.138	0.697 ± 0.115	0.709 ± 0.101	0.848 ± 0.114	0.447 ± 0.173
CTNet	73.40 ± 7.26	0.710 ± 0.072	0.826 ± 0.146	0.768 ± 0.079	0.814 ± 0.209	0.654 ± 0.143	0.734 ± 0.073	*0.741 ± 0.116*	0.708 ± 0.071	0.725 ± 0.079	*0.860 ± 0.106*	0.468 ± 0.145
EEGNeX	*74.18 ± 9.68*	**0.772 ± 0.072**	0.798 ± 0.175	*0.785 ± 0.079*	0.719 ± 0.299	**0.765 ± 0.155**	*0.742 ± 0.097*	0.701 ± 0.185	**0.750 ± 0.068**	*0.726 ± 0.115*	**0.874 ± 0.091**	*0.484 ± 0.194*
PDC-Net	**76.76 ± 11.90**	0.734 ± 0.130	**0.847 ± 0.131**	**0.790 ± 0.116**	*0.882 ± 0.107*	0.653 ± 0.196	**0.768 ± 0.119**	**0.795 ± 0.099**	0.726 ± 0.154	**0.760 ± 0.125**	0.855 ± 0.110	**0.535 ± 0.238**
(**b**)												
Model			**Δ** **Acc (%)**		**95% CI of ΔAcc**		**Wilcoxon W**		**Holm-Adjusted *p***		***r*** **_rb_**	
SVM			17.50		[14.80, 20.10]		7.0		0.005		0.897	
RF			13.36		[10.50, 16.20]		4.0		0.002		0.941	
XGBoost			14.06		[11.10, 17.00]		2.0		0.001		0.971	
EEGNet			4.53		[−0.18, 9.32]		18.0		0.023		0.735	
DeepConvNet			10.04		[6.80, 13.20]		4.0		0.002		0.941	
ShallowConvNet			4.22		[1.49, 7.07]		12.0		0.011		0.824	
TSception			5.55		[1.36, 10.01]		8.0		0.005		0.882	
EEGConformer			4.42		[0.31, 8.70]		16.0		0.021		0.765	
CTNet			3.36		[−1.27, 8.28]		30.0		0.101		0.559	
EEGNeX			*2.58*		[−1.18, 6.37]		32.0		0.107		0.529	

**Table 3:** Computational characteristics and decoding accuracy of the evaluated deep-learning models under the offline GPU setting. Bold and italic values indicate the best and second-best results, respectively; lower values are preferred for Parameters, MACs, FLOPs, and Latency, whereas higher values are preferred for Accuracy.

Model	Parameters (M)	MACs (M)	FLOPs (M)	Latency (ms/Sample)	Acc (%)
EEGNet	**0.002**	**0.890**	**1.780**	*0.401 ± 0.094*	72.23 ± 9.07
DeepConvNet	0.266	10.574	21.147	0.720 ± 0.272	66.72 ± 10.01
ShallowConvNet	*0.008*	3.779	7.557	**0.361 ± 0.075**	72.54 ± 9.88
TSception	0.295	*2.927*	*5.854*	0.517 ± 0.172	71.21 ± 11.11
EEGConformer	0.608	10.327	20.655	3.086 ± 0.737	72.34 ± 8.65
CTNet	0.152	7.607	15.214	3.405 ± 1.086	73.40 ± 7.26
EEGNeX	0.055	32.371	64.742	0.600 ± 0.157	*74.18 ± 9.68*
PDC-Net	0.939	3.516	7.109	0.460 ± 0.089	**76.76 ± 11.90**

**Table 4:** Probe-control and cross-condition generalization results. MP and NP denote the matched-probe and no-probe conditions, respectively; the arrow indicates the direction from the training condition to the test condition. Values are presented as the mean ± standard deviation across the 16 held-out participants. MP → MP was treated as the reference condition. ΔAcc was calculated as the accuracy of MP → MP minus that of each control condition. The 95% confidence intervals were calculated from participant-level paired accuracy differences, and the *p*-values were adjusted across the three prespecified comparisons using the Holm procedure.

Training → Test	Acc (%)	ΔAcc (%)	95% CI of ΔAcc	Pre	Rec	F1	AUC	Kappa	Holm-Adjusted *p*
MP → MP	76.76 ± 11.90	—	—	0.790 ± 0.116	0.768 ± 0.119	0.760 ± 0.125	0.855 ± 0.110	0.535 ± 0.238	—
NP → NP	74.38 ± 10.06	2.38	[−0.34, 5.38]	0.732 ± 0.093	0.744 ± 0.101	0.719 ± 0.129	0.754 ± 0.120	0.488 ± 0.201	0.183
MP → NP	71.33 ± 8.38	5.43	[1.47, 8.01]	0.720 ± 0.082	0.713 ± 0.084	0.711 ± 0.085	0.727 ± 0.095	0.427 ± 0.168	0.019
NP → MP	70.27 ± 9.93	6.49	[3.46, 9.28]	0.739 ± 0.087	0.703 ± 0.099	0.686 ± 0.120	0.725 ± 0.104	0.405 ± 0.199	0.012

## Data Availability

The de-identified data presented in this study are available from the corresponding author upon reasonable request, subject to applicable institutional and ethical requirements. The participant-partition rule and the preprocessing, sample-selection, model-configuration, and hyperparameter settings required to reproduce the reported analyses are described in Section 3.1, Section 3.2, Section 3.3 and Section 3.4 and Supplementary Tables S1–S5.

## Supplementary Materials

The following supporting information can be downloaded at https://www.mdpi.com/article/10.3390/brainsci16090915/s1, Table S1: Participant-level trial and sample accounting for all experimental conditions; Table S2: Trial-level probe and virtual-probe schedules, acquisition details, eligibility decisions, and final paired-trial selections; Table S3: Combined artifact-robustness analysis of PDC-Net and participant-level artifact-burden accounting; Table S4: Predefined behavioral quality-control criteria, participant-specific calibration thresholds, and behavioral trial-exclusion accounting; Table S5: Principal fixed settings and hyperparameter search spaces for the traditional machine-learning and deep-learning models.

## Abbreviations

The following abbreviations are used in this manuscript:

BCI	Brain–computer interface
EEG	Electroencephalography
BN	Batch Normalization
CGMI	Cross-Branch Gated Mamba Interaction
CNN	Convolutional Neural Network
CTNet	Convolutional Transformer Network
FFN	Feed-Forward Network
GAP	Global Average Pooling
GELU	Gaussian Error Linear Unit
ICA	Independent Component Analysis
IRB	Institutional Review Board
LOSO	Leave-One-Subject-Out
ML	Machine Learning
PDC-Net	Prefrontal Dual-channel Network
PSD	Power Spectral Density
RBF	Radial Basis Function
ReLU	Rectified Linear Unit
RF	Random Forest
RT	Reaction Time
RTV	Reaction Time Variability
SSM	State Space Model
SVM	Support Vector Machine
TRAB	Temporal Representation Adaptation Block
t-SNE	t-distributed Stochastic Neighbor Embedding
VSA	Visual Sustained Attention
VIDC	Visual Internally Directed Cognition
XGBoost	Extreme Gradient Boosting
ZOH	Zero-Order Hold
EMG	Electromyography
